# Tau is required for the function of extrasynaptic NMDA receptors

**DOI:** 10.1038/s41598-019-45547-8

**Published:** 2019-06-24

**Authors:** Noemí Pallas-Bazarra, Jonathan Draffin, Raquel Cuadros, José Antonio Esteban, Jesús Avila

**Affiliations:** 1 Centro de Biología Molecular Severo Ochoa (CBMSO) CSIC-UAM, Madrid, Spain; 2 Network Center for Biomedical Research in Neurodegenerative Diseases (CIBERNED), Madrid, Spain

**Keywords:** Molecular neuroscience, Synaptic transmission

## Abstract

Tau is a microtubule-associated neuronal protein found mainly in axons. However, increasing evidence indicates that it is also present in dendrites, where it serves as an essential mediator of synaptic NMDA (N-methyl-D-aspartate) receptor-dependent excitotoxicity. Of note, NMDA receptors can also be found outside synapses in the plasma membrane, and activation of extrasynaptic NMDA receptors has been shown to be more linked to excitotoxicity than the activation of synaptic ones. Little is known about the role of Tau in the activity of extrasynaptic NMDA receptors. Thus, we have used a Tau knockout mouse model (Tau^−/−^ mice) to analyze the consequences of Tau absence in extrasynaptic NMDA receptor activity. We demonstrate that absence of Tau leads to a decrease in functional extrasynaptic NMDA receptors in the hippocampus. We propose that this impairment in extrasynaptic NMDA receptor activity may contribute to the well-known neuroprotective effect associated with Tau deficiency under pathological conditions.

## Introduction

Alzheimer’s disease (AD) is characterized by the presence of two aberrant structures, namely senile plaques (composed by aggregates of beta amyloid (Aβ) peptide) and neurofibrillary tangles (composed by the neuronal Tau protein). On the basis of genetic analysis of patients with familiar AD, it has been proposed that accumulation of Aβ aggregates is the initial event of neurodegeneration. However, increasing evidence supports the notion that Tau is essential for Aβ-induced detrimental effects in neurons^1–3^. In this regard, it has been suggested that dendritic Tau is required to drive NMDA receptor-mediated excitotoxicity induced by Aβ^4^.

NMDA receptors are cation channels gated by the neurotransmitter glutamate. They are composed by tetrameric complexes containing two GluN1 subunits and two GluN2 subunits (GluN2A and/or GluN2B), the latter vary in their regional expression profiles, desensitization kinetics, pharmacological properties and downstream signaling pathways^5^. NMDA receptors are located mainly at glutamatergic synapses, being essential mediators of synaptic transmission and of many forms of synaptic plasticity. Glutamate-induced activation of synaptic NMDA receptors is typically associated with pro-survival signaling pathways; however, in response to Aβ binding, they can also trigger neuronal death^5^. The mechanisms underlying this toxicity are not fully understood, but Ittner and colleagues demonstrated that Tau is necessary for the transport of the tyrosine kinase Fyn to synapses, where it phosphorylates the GluN2B subunit of NMDA receptors. GluN2B phosphorylation enhances the interaction of these receptors with the postsynaptic scaffold protein PSD95, being stable NMDA receptor-PSD95 complexes necessary to drive Aβ induced excitotoxicity^4^.

Interestingly, in addition to their synaptic location, NMDA receptors can also be found outside synapses. Extrasynaptic NMDA receptors have received much less attention than synaptic ones, but they appear to be typically activated by elevated levels of ambient glutamate, and they lead to the activation of cell death pathways^5,6^. Of note, Aβ toxicity, through its interaction with extrasynaptic NMDA receptors, is much higher than that reported for synaptic receptors^5^.

Intriguingly, genetic deletion of Tau appears to protect animals against a variety of neurotoxic insults associated with the overactivation of extrasynaptic NMDA receptors, such as stroke^7^, epileptic seizures^8,9^, acute stress^10^ and traumatic brain injury^11^. In this context, we sought to determine whether Tau protein is involved in the activity of extrasynaptic NMDA receptors.

## Results

Dendritic Tau has been indirectly associated to synaptic NMDA receptor function^4^. However, little is known about its contribution to the function of extrasynaptic NMDA receptors. Thus, we analyzed possible differences in the composition and/or in the function of NMDA receptors in WT and Tau^−/−^ mice.

### Tau protein is not involved in the regulation of the levels of NMDA receptors in the hippocampus *i****n vivo***

**Figure 1:**
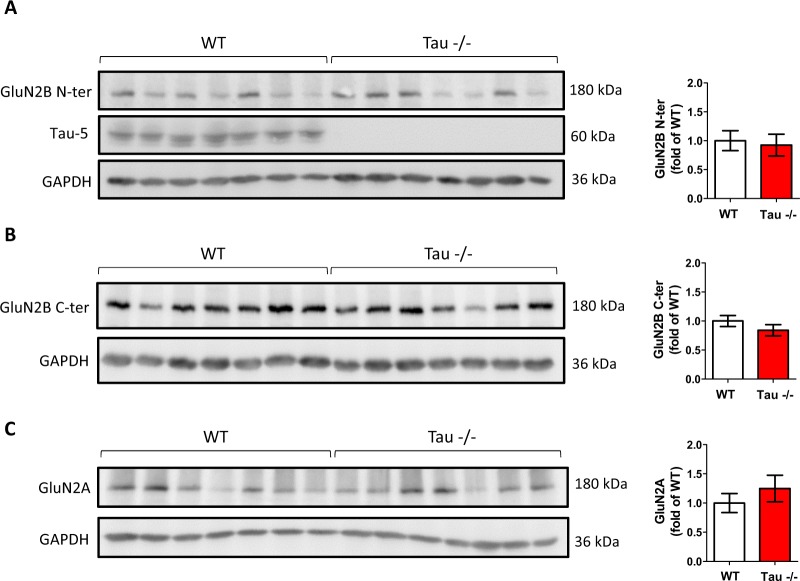
Absence of Tau does not alter NMDA receptor levels in the hippocampus. Western blot (WB) analysis of the levels of Tau and of GluN2B subunits of NMDA receptors with an antibody directed against its N-terminus (**A**) or its C-terminus (**B**), and of GluN2A subunits of NMDA receptors (**C**), from hippocampal extracts of WT and Tau^−/−^ mice. Quantifications of GluN2B N-ter, GluN2B C-ter and GluN2A levels are shown on the right. Tau protein is not detected in Tau^−/−^ mice, and the levels of GluN2B N-ter, GluN2B C-ter and GluN2A subunits are not affected by the absence of Tau. Graphs show mean ± SEM. N = 7 mice per genotype. GAPDH was used as loading control. Blots were cropped to improve the clarity of the presentation. Full-length blots are presented in Supplementary Fig. 5.

Specific NMDA receptor subunits are not confined to specific localizations in the plasma membrane. However, it has been proposed that GluN2B-containing receptors are more mobile than those containing GluN2A, thus contributing to an enrichment in the former at extrasynaptic sites and in the latter at synaptic sites^12^. Thus, in order to examine a possible role of Tau protein in the expression and/or distribution of NMDA receptors, we analyzed the levels of GluN2 subunits in the hippocampi of WT and Tau^−/−^ mice. Western blot analysis revealed no differences in the levels of GluN2B subunits recognized with an antibody directed against the N-terminal domain of the protein (GluN2B N-ter) (t = 0.294; p = 0.774) (Fig. 1A). Of note, this N-terminal domain is always present in GluN2B-containing NMDA receptors. In contrast, the C-terminal domain of GluN2B subunits can be cleaved in extrasynaptic pools of the receptor^12^. In such cases, GluN2B protein would not be expected to be recognized by an antibody directed against this C-terminus (GluN2B C-ter), a feature that could help to distinguish between different pools of NMDA receptors. However, no differences were found either between WT and Tau^−/−^ mice when the GluN2B C-ter antibody was used (t = 1.181; p = 0.260) (Fig. 1B). Similarly, no differences were found in the levels of GluN2A subunits (t = −0.889; p = 0.391) (Fig. 1C).

### Absence of Tau protein does not alter post-translational GluN2B modifications

**Figure 2:**
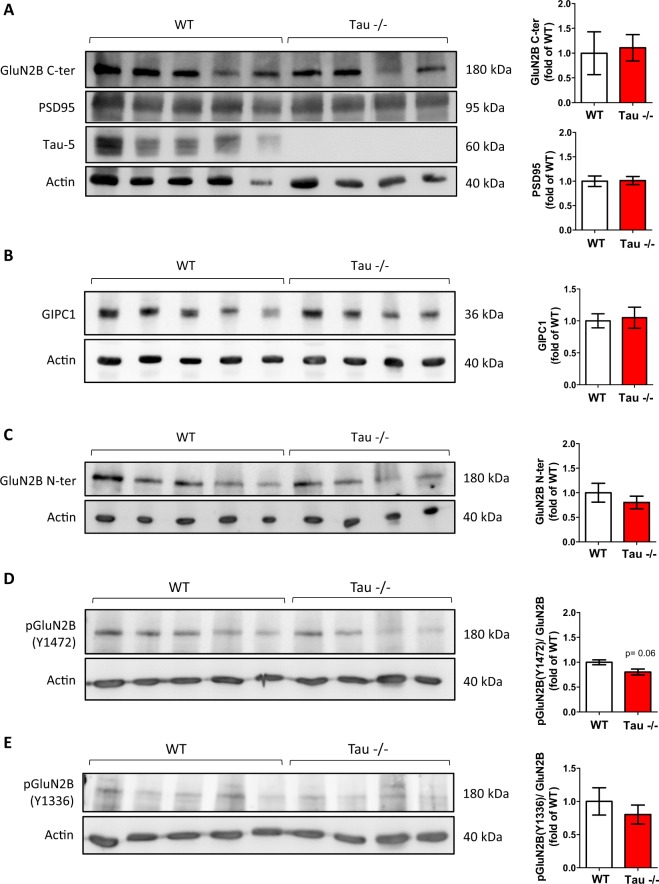
Absence of Tau does not alter NMDA receptor levels in synaptosome-enriched hippocampal fractions. Western blot (WB) analysis of the levels of Tau, PSD95, GIPC1 and GluN2B receptor subunits of NMDA receptors in synaptosome-enriched fractions obtained from the hippocampi of WT and Tau^−/−^ mice. (**A**) Tau is not present in synaptosome-enriched fractions obtained from the hippocampi of Tau^−/−^ mice, and this absence does not affect the levels of PSD95 or those of GluN2B subunits of NMDA receptors detected with an antibody directed against its C-terminus. Quantifications are shown on the right. (**B**) The levels of GIPC1 are not altered in Tau^−/−^ synaptosome-enriched fractions as compared to WT ones. Quantification is shown on the right. (**C**) The levels of GluN2B subunits of NMDA receptors detected with an antibody directed against its N-terminus are not altered in synaptosome-enriched fractions of Tau^−/−^ mice as compared to WT ones. Quantification is shown on the right. (**D**) The levels of GluN2B subunits of NMDA receptors phosphorylated at Y1472 (a phosphorylation occurring mainly at synaptic sites) tend to decrease in synaptosome-enriched fractions of Tau^−/−^ mice as compared to WT ones. Quantification is shown on the right. (**E**) The levels of GluN2B subunits of NMDA receptors phosphorylated at Y1336 (a phosphorylation occurring mainly at extrasynaptic sites) are not significantly altered in synaptosome-enriched fractions of Tau^−/−^ mice as compared to WT ones. Quantification is shown on the right. Graphs show mean ± SEM. N WT = 5 mice; N Tau^−/−^ = 4 mice. The levels of pGluN2B(Y1472) and pGluN2B(Y1336) were calculated related to the levels of GluN2B N-ter. Actin was used as loading control. Blots were cropped to improve the clarity of the presentation. Full-length blots are presented in Supplementary Fig. 5.

With the aim to fine-tune the analysis of the effects of Tau absence on NMDA receptor distribution, we next examined synaptosome-enriched fractions obtained from the hippocampi of WT and Tau^−/−^ mice. The absence of Tau did not alter the levels of PSD95 (U = 7.000; p = 0.556), the main scaffold synaptic protein (Fig. 2A). Likewise, no differences were found in the levels of GIPC (U = 10.000; p = 1.000) (Fig. 2B), an alternative scaffolding protein that preferentially stabilizes extrasynaptic NMDA receptors^13^. Similarly, Tau absence did not lead to differences in the levels of GluN2B recognized either in its C-terminus (U = 8.000; p = 0.730) or N-terminus domain (U = 8.000; p = 0.730) (Fig. 2A,C).

GluN2B are thought to be the most mobile and widespread distributed subunits of NMDA receptors^12^. In this regard, posttranslational modifications, such as tyrosine phosphorylation, have been related to GluN2B cell surface mobility. Thus, we next analyzed the levels of pGluN2B(Y1472), a phosphorylation found mainly on synaptic GluN2B subunits, and pGluN2B(Y1336), a phosphorylation found mainly on extrasynaptic GluN2B subunits^14^, in synaptosome-enriched fractions of WT and Tau^−/−^ mice. No differences were detected in the levels of either of these phosphorylated forms relative to total GluN2B levels, although pGluN2B(Y1472) tended to decrease in Tau^−/−^ mice (U pGluN2B(Y1472) = 2.000; p = 0.063; U pGluN2B(Y1336) = 9.000; p = 0.905) (Fig. 2D,E). Moreover, since phosphorylation at S1480 of GluN2B subunits mediates NMDA receptor internalization from synapses by disrupting their interaction with PSD95^15^, we evaluated the levels of pGluN2B(S1480) in WT and Tau^−/−^ synaptosome-enriched fractions. No differences were found either in this parameter, although it tends to be increased in Tau^−/−^ mice (U = 0.000; p = 0.05) (Supplementary Fig. 1).

**Figure 3:**
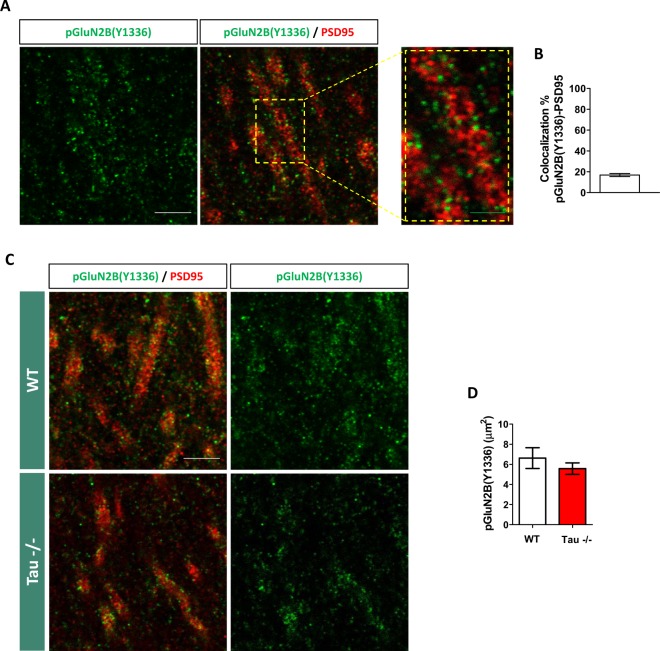
The distribution of GluN2B phosphorylated at Y1336, a post-translational modification occurring mainly at extrasynaptic sites, is not affected in the CA1 region of Tau^−/−^ mice. (**A**) Representative image of CA1 hippocampal region of a WT mouse labeled with pGluN2B(Y1336) (green channel) and PSD95 (red channel) antibodies, as well as a high-power magnification showing that pGluN2B(Y1336) labeling is located close to but not overlapping PSD95 labeling. (**B**) Quantification of the percentage of co-localization between pGluN2B(Y1336) and PSD95 in WT mice. (**C**) Representative images of WT and Tau^−/−^ CA1 hippocampal regions labeled with pGluN2B(Y1336) (green channel) and PSD95 (red channel) antibodies. (**D**) Quantification of the area occupied by pGluN2B(Y1336). Tau absence does not alter the area occupied by pGluN2B(Y1336) in the CA1 region of the hippocampus. Graphs show mean ± SEM. N = 5 mice per genotype. White scale bar 5 µm. Green scale bar 2 µm. Brightness and contrast of representative confocal microscopy images shown in the figure were minimally adjusted in order to improve visualization.

To further evaluate the involvement of Tau in extrasynaptic NMDA receptor distribution, we analyzed by immunohistochemistry the area occupied by pGluN2B(Y1336) in the hippocampi of WT and Tau^−^/ mice. Figure 3A shows representative images of pGluN2B(Y1336) and PSD95, the latter a marker of synaptic sites, in the CA1 region of the hippocampi of WT animals. Consistent with the association of Y1336 phosphorylation mainly with extrasynaptic GluN2B subunits, the percentage of colocalization between pGluN2B(Y1336) and PSD95 was relatively low: 16.82 ± 1.43% (Fig. 3B). Figure 3C shows representative images of the area occupied by pGluN2B(Y1336) in the CA1 region of WT and Tau^−/−^ mice. No significant differences caused by the absence of Tau were found in this parameter (U = 1181.000; p = 0.758) (Fig. 3D). Strikingly, the percentage of colocalization between pGluN2B(Y1336) and PSD95 in Tau^−/−^ animals was reduced as compared to WT ones (U = 828.000; p = 0.004). However, this effect was found to be the consequence of a decrease in the area occupied by PSD95 in Tau^−/−^ mice (U = 729.000; p = 0.001) (Supplementary Fig. 2).

In summary, these results suggest that Tau protein does not affect the total levels or the subcellular distribution of NMDA receptor subunits in the hippocampus.

### Tau^−/−^ neurons lack extrasynaptic NMDA receptor currents

Alterations in the dynamics of postsynaptic densities related to impaired structural plasticity were recently described in Tau^−/−^ granule neurons^10^. Importantly, this lack of plasticity could involve decreased mobility of NMDA receptors at synapses, which in turn has been related to defective long-term depression (LTD)^16^. Actually, Tau^−/−^ neurons show impaired LTD^17^, and Tau phosphorylation at S396 has been suggested to be involved in such type of synaptic plasticity^18^. According to that, we found pTau(S396) in synaptosome-enriched fractions of WT animals (Supplementary Fig. 3). Thus, it is possible that absence of Tau results in a decrease in the amount of extrasynaptic NMDA receptors, arising from a deficient dendritic spine turnover^12^, which cannot be detected by biochemical approaches due to the low percentage of such receptors compared to synaptic ones^19,20^. In order to test this hypothesis, we evaluated next potential functional differences in WT and Tau^−/−^ neurons by using electrophysiological recordings.

**Figure 4:**
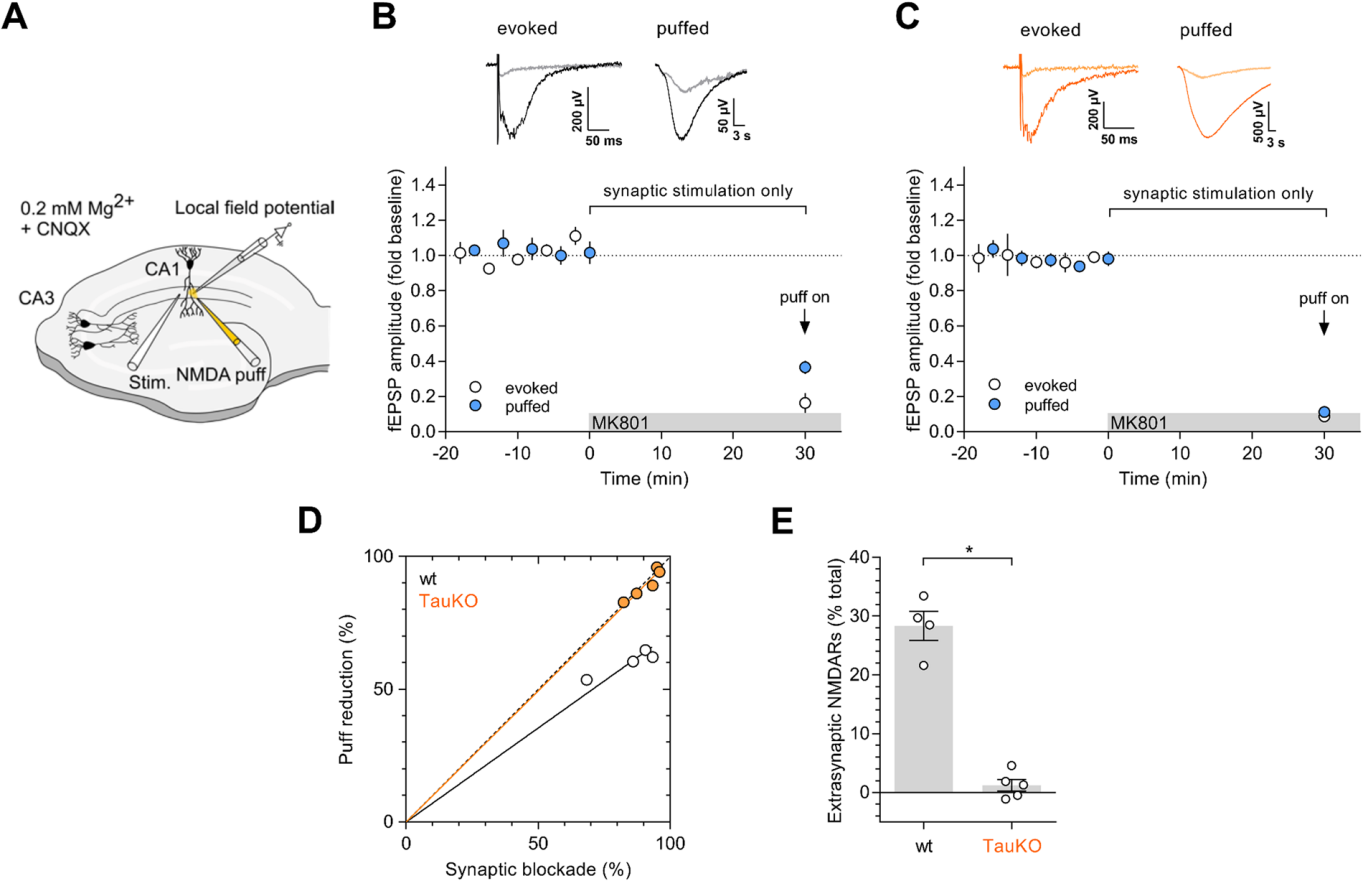
Electrophysiological detection of extrasynaptic NMDA receptors in the CA1 region of wild-type and Tau^−/−^ mice. (**A**) Cartoon representation of the experimental configuration for local field potential recordings in the CA1 region of hippocampal slices using electrical stimulation of CA3 Schaffer collaterals (for synaptic responses) and a glass pipette to deliver local NMDA puffs (for synaptic plus extrasynaptic responses). Currents from NMDA receptors are isolated by blocking AMPA receptors with CNQX and in the presence of a low concentration of Mg^2+^. (**B**,**C**) Time course of NMDA receptor-mediated responses from WT (B) and Tau^−/−^ (**C**) slices with alternating synaptic (white symbols) and puff (blue symbols) stimulation. MK801 is added to the perfusion solution at t = 0, as indicated (gray bar). At this point, puffed stimulation is stopped, and it is resumed only once inhibition of synaptic responses has stabilized (indicated with an arrow). (**D**) Scatter plot for the relative inhibition of electrical (synaptic) and puff (synaptic plus extrasynaptic) responses after MK801 incubation for slices from WT (white symbols) and Tau^−/−^ (orange symbols) animals. Dotted diagonal line represents identical inhibition of both responses. Points below the diagonal indicate stronger inhibition of the synaptic response as compared to the puff response. (**E**) Calculation of the relative contribution of extrasynaptic receptors to the puff response from the data shown in (D), as described in (Papouin et al., Cell 2012 Aug 3;150(3):633-46).

To isolate responses from extrasynaptic receptors, we used a strategy based on blocking synaptic responses with the irreversible, use-dependent NMDA receptor blocker MK-801 during synaptic stimulation^19,20^. Essentially, baseline NMDA receptor responses from hippocampal slices are recorded, alternating Schaffer collateral synaptic stimulation and local NMDA puffs on the *stratum radiatum* (see scheme in Fig. 4A). The NMDA puff is expected to elicit a combined response from both synaptic and extrasynaptic receptors. The contribution from synaptic receptors is then removed by continuing Schaffer collateral stimulation in the presence of MK-801, while puffed stimulation is stopped. Once the inhibition of synaptic responses is stabilized, the puffed stimulation is resumed. The relative inhibition of the new puffed response is expected to be lower than that of the synaptic response, as extrasynaptic receptors should not have been blocked during the MK801 incubation (see result from WT mice in Fig. 4B). By comparing the extent of inhibition of the electrical and puffed responses, the contribution of the extrasynaptic receptors can be calculated (Fig. 4D, WT)^19^. This contribution was about 30% in WT mice (Fig. 4E), which is in good agreement with previous calculations using this method^19,20^, and also with morphological calculations using immunogold electron microscopy^21^. Strikingly, the extent of MK-801 inhibition was virtually identical for the synaptic and puffed responses in the case of Tau^−/−^ slices (Fig. 4C, points on the diagonal line in Fig. 4D). These numbers indicate a close to null contribution of extrasynaptic receptors to the puffed response in the absence of Tau (Fig. 4E).

Therefore, these electrophysiological recordings reveal the virtual absence of functional extrasynaptic NMDA receptors in CA1 neurons of Tau^−/−^ mice.

## Discussion

Amyloid-induced toxicity has been proposed to occur through the interaction of the peptide with NMDA receptors. Compared to AMPA receptors, NMDA receptors show a more stable presence at the cell membrane, thereby facilitating the entry of calcium, which can be toxic over certain levels^22^. Interestingly, NMDA receptors can be found both inside and outside synapses^21^, the latter being more associated with the activation of cell death pathways^5^.

Here, we report for the first time electrophysiological recordings showing a decreased functionality of extrasynaptic NMDA receptors in the hippocampus of Tau^−/−^ mice. Of note, absence of Tau has been extensively associated to neuroprotection against NMDA receptor-dependent excitotoxicity^23^. Traditionally, this neuroprotection has been related to a dendritic role of Tau in the functionality of synaptic NMDA receptors^4^. However, given the strong association of extrasynaptic NMDA receptors with cell death pathways, we propose the impairment in extrasynaptic NMDA receptor functionality as an additional neuroprotective mechanism led by Tau absence.

**Figure 5:**
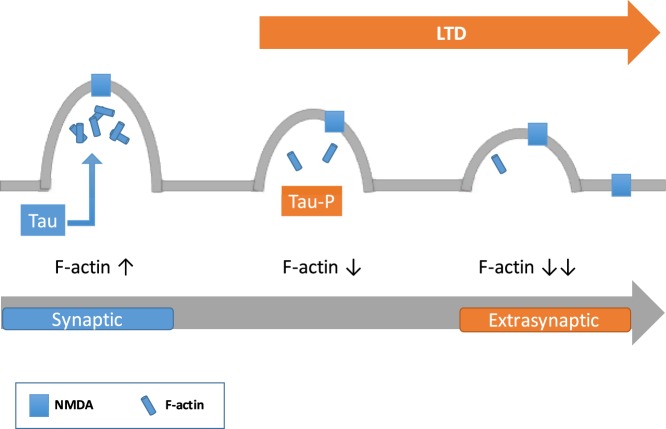
Tau may regulate NMDA receptor trafficking through actin depolymerization. As discussed in the text, Tau phosphorylated could facilitate F-actin depolymerization in the spine in a direct or indirect way, and as consequence of that, synapytic NMDA receptors could move to extrasynaptic regions. This effect may not take place in Tau^−/−^ mice.

NMDA receptors can laterally diffuse between synaptic and extrasynaptic sites, although the mechanisms underlying this mobility are not fully understood. In this regard, GluN2B subunits are thought to be more mobile, widespread and less confined to synapses than GluN2A subunits^5^. Indeed, posttranslational modifications of GluN2B have been related to specific NMDA receptor localizations^14^. Phosphorylation of GluN2B at Y1472 promotes the interaction of the subunit with scaffold proteins like PSD95^4^, thus stabilizing NMDA receptors in the postsynaptic density^24^. Accordingly, phosphorylation of GluN2B at Y1472 is decreased in extrasynaptic NMDA receptors, the phosphorylation at GluN2B Y1336 being predominant in these receptors^14,21^. Thus, the levels of GluN2B phosphorylation at Y1472 and Y1336 could serve as indirect markers of synaptic and extrasynaptic NMDA receptors, respectively. However, we did not find differences in Y1472 or Y1336 GluN2B phosphorylation in response to the absence of Tau. Thus, it cannot be assumed a decrease in the levels of extrasynaptic receptors in Tau^−/−^ animals based on this criteria. Nevertheless, the mechanisms that regulate NMDA receptor subcellular localization and function includes a wide range of protein-protein interactions, phosphorylation, palmitoylation, ubiquitination and receptor proteolytic cleavage^12^. Thus, additional experiments would be required to further analyze the involvement of Tau in the localization of NMDA receptors at synaptic and extrasynaptic sites. Noteworthy, there is a well-known association of Tau with actin^16,18,25^, and actin is closely related to NMDA receptor localization and function: depolymerization of actin filaments decrease NMDA receptor signaling^26^; changes in actin dynamics are required for long-term plasticity and synaptic function^27,28^; and alterations of the actin cytoskeleton may facilitate the lateral diffusion of NMDA receptors from synaptic to extrasynaptic sites^29^. In line with this, Tau phosphorylated at serine 396 has been related to the induction of long-term depression (LTD)^17^, and as a result of LTD actin cytoskeleton ultimately undergoes depolymerization^30^. As a consequence, synaptic NMDA receptors could be moved to extrasynaptic regions^12^ (Fig. 5). Thus, Tau may be required to facilitate changes in the actin cytoskeleton surrounding NMDA receptors to facilitate their lateral diffusion, and in the absence of Tau, less functional extrasynaptic NMDA receptors could be found.

Altogether, our results reveal that lack of Tau protein impairs the functionality of extrasynaptic NMDA receptors. Given the pivotal role of this type of NMDA receptors in the activation of cell death pathways^5,6^, this decreased functionality may contribute to the well-known neuroprotection conferred by the absence of Tau^1,2,4,10,31^. However, it is important to note that Tau deletion is not always neuroprotective. For example, acute knockdown of Tau at the hippocampus may cause memory deficits^32^, Tau deletion can promote brain insulin resistance^33^, and absence of Tau results in impaired plasticity of newborn granule cells^10^. In line with this, absence or pathological modifications of Tau have been associated with impaired LTP^34–37^, which in turn is closely related to synaptic functionality. Thus, absence of Tau can be beneficial or detrimental depending on the nature of the stimuli and the cell processes affected. In this regard, the novel effect of Tau on extrasynaptic NMDA receptor functionality reported here could represent a more specific target for therapies based on the neuroprotection conferred by the removal of Tau.

In summary, we have used biochemical and electrophysiological analyses to study the involvement of Tau protein in the functionality of extrasynaptic NMDA receptors. Based on electrophysiological analysis, we found that Tau is required for the function of these receptors. Since that function is related to Aβ neuronal toxicity, we propose that such impairment in extrasynaptic NMDA receptor activity may contribute to the neuroprotective effect of Tau deficiency found in the presence of Aβ.

## Methods

### Animals

Tau^−/−^ mice were generated as previously described^38^. Heterozygous (Tau^+/−^) mice were crossed in order to obtain homozygous Tau knockout mice (Tau^−/−^) and control littermates (WT). Mice were housed under standard laboratory conditions in a specific pathogen-free colony facility at the *Centro de Biología Molecular “Severo Ochoa”* (CBMSO). Housing conditions were in accordance with European Community Guidelines (directive 86/609/EEC), and animals were handled complying with European and local animal care protocols. Animal experiments received the approval of the CBMSO Ethics Committee and the National Ethics Committee (PROEX 291/15). Both male and female mice were included in this study. Depending on the experiment, 4–7 animals per genotype were used. They were 4 months old at the time of sacrifice for biochemical and immunohistochemical analyses, and in a range of 7–9 months old for electrophysiological recordings.

### Sacrifice and tissue processing

Animals were fully anesthetized by an intraperitoneal injection of pentobarbital (EutaLender, 60 mg/kg) and perfused intracardially with 0.9% saline. For immunohistochemical analysis, brains were removed and fixed overnight in 4% paraformaldehyde (PFA). Sagittal sections (50 µm thick) were obtained on a Leica VT1200S vibratome, and series of sections comprising every 8th section were generated. For biochemical analysis of tissue extracts, hippocampi were dissected and preserved at −80 °C until processing. For biochemical analysis of synaptosome-enriched fractions, hippocampi were dissected, and immediately subjected to subcellular fractionation.

For electrophysiological experiments, coronal acute hippocampal slices (300 µm thick) were prepared. Mice were briefly anesthetized using dry ice sublimated with water, and the brains were removed and placed in partially frozen Ca^2+^-free dissection medium (10 mM D-Glucose, 4 mM KCl, 26 mM NaHCO_3_, 234 mM sucrose, 5 mM MgCl_2_, 1:1000 Phenol Red) saturated with 5% CO_2_/95% O_2_. Coronal slices were cut using a Leica VT1200S vibratome and recovered for 1 h at 32 °C in a submersion-type incubation chamber containing artificial cerebrospinal fluid (ACSF; 119 mM NaCl, 2.5 mM KCl, 2.5 mM CaCl_2_, 1.2 mM MgCl_2_, 26 mM NaHCO_3_, 1 mM NaH_2_PO_4_, 11 mM glucose; pH 7.4). Subsequently, the incubation temperature was lowered to 25 °C, at which point the slices were considered ready for experimentation.

### Subcellular fractionation

Subcellular fractionation of hippocampal tissue was performed following the protocol described in^39^, with minor modifications. Briefly, hippocampi were dissected and homogenized in ice-cold buffer containing 0.32 M sucrose, 1 mM HEPES, 1 mM MgCl_2_, 1 mM EDTA and 1 mM NaHCO_3_. The homogenized tissue was centrifuged at 1000 *g*, 4 °C for 10 min, and the resulting supernatant was centrifuged again at 13000 *g*, 4 °C for 15 min to obtain a crude synaptosome-enriched fraction in the pellet. This pellet was then resuspended in buffer containing 75 mM KCl and 1% Triton X-100 and centrifuged at 100000 *g*, 4 °C for 90 min. The resulting pellet, enriched in membrane-associated proteins, was resuspended in 20 mM HEPES to obtain a final synaptosome-enriched fraction. Protein content was determined by the Pierce® BCA Protein Assay kit (Thermo Scientific), and samples were processed for Western blot analysis. All buffers used in this protocol contained phosphatase inhibitor cocktail (PhosSTOP, Sigma-Aldrich) and protease inhibitor cocktail (Complete, Roche).

The rate of enrichment in membrane-associated proteins was determined by analyzing the levels of PSD95, which is a suitable synaptosomal biochemical marker^40,41^, in final synaptosome-enriched fractions (20 µg of total protein) compared to hippocampal extracts (20 µg of total protein) obtained from WT and Tau^−/−^ mice (PSD95 fold change WT = 2.5 ± 0.07; PSD95 fold change Tau^−/−^ = 1.8 ± 0.2) (Supplementary Fig. 4).

### Western blot

Hippocampal extracts for Western Blot analysis were prepared in RIPA buffer consisting of 50 mM pH 7.4 Tris-HCl, 1% NP-40, 150 mM NaCl, 1 mM EDTA, 0.25% sodium deoxycholate, phosphatase inhibitors (1 mM NaF, 1 mM Na_3_VO_4_ and 1 µM okadaic acid) and protease inhibitor cocktail (Complete, Roche). Samples were homogenized at 4 °C, and protein content was determined by the Pierce® BCA Protein Assay kit (Thermo Scientific). Total protein—25 µg for the analysis of hippocampal extracts and 20 µg for the analysis of synaptosome-enriched fractions (see above)—was electrophoresed on 8% SDS-PAGE gel and transferred to a nitrocellulose membrane (Schleicher and Schuell). Membranes were then blocked with 5% bovine serum albumin in TBS with 0.1% Tween-20 and incubated at 4 °C overnight with the following primary antibodies: mouse anti-Tau5 (Calbiochem, 1:1000); guinea pig anti-PSD95 (Synaptic Systems, 1:1000); rabbit anti-GIPC1 (Abcam, 1:1000); mouse anti GluN1 (Millipore, 1:1000); rabbit anti-GluN2B C-ter (Millipore, 1:1000); rabbit anti-GluN2B N-ter (Phosphosolutions, 1:1000); rabbit anti-GluN2A (Phosphosolutions, 1:1000); rabbit anti-pGluN2B(Y1472) (Thermo Scientific, 1:500); rabbit anti-pGluN2B(Y1336) (Phosphosolutions, 1:1000); rabbit anti-pGluN2B(S1480) (Phosphosolutions, 1:1000); rabbit anti-pTau(S396) (Life Techn., 1:1000); mouse anti-GAPDH (Abcam, 1:2000); and mouse anti-β actin (Sigma, 1:2000). Secondary antibodies goat anti-rabbit (Dako, 1:1000), goat anti-mouse (Dako, 1:1000), and goat anti-guinea pig (Dako, 1:1000), followed by ECL detection reagents (Amersham), were used for immunodetection.

### Immunohistochemistry

Immunohistochemistry was performed as previously described^42^. Briefly, slices were pre-incubated in phosphate buffer with 1% Triton X-100 and 1% bovine serum albumin and then incubated with the primary antibodies guinea pig anti-PSD95 (Synaptic Systems, 1:1000) and rabbit anti-pGluN2B(Y1336) (Phosphosolutions, 1:400) at 4 °C for 48 h. To detect the binding of primary antibodies, goat Alexa 555 anti-guinea pig (ThermoFisher, 1:1000) and donkey Alexa 488 anti-rabbit (ThermoFisher, 1:1000) were used. Sections were counterstained for 10 min with DAPI (Merck, 1:5000) to label nuclei.

### Analysis of the fluorescence intensity of extrasynaptic-enriched GluN2B subunits (pGluN2B Y1336) in the CA1 region of the hippocampus

Single plane images were acquired in a LSM710 Zeiss confocal microscope (63x oil immersion objective; XY dimensions: 24.1 μm). Five animals per genotype were used in this analysis, and ten images of randomly selected regions of the CA1 region of the hippocampus per animal were obtained from the sections comprising the series. To analyze all the images, an invariant threshold was applied to the green (pGluN2B Y1336) and red (PSD95) channels using Metamorph software (Molecular Devices), and the area over the threshold was automatically calculated. The percentage of colocalization between pGluN2B(Y1336) and PSD95 was also calculated by the software.

### Electrophysiological recordings

Electrophysiological experiments to determine the extrasynaptic proportion of functional NMDA receptors were loosely based on the procedure described in^19^. Briefly, electrically evoked field excitatory postsynaptic potentials (fEPSPs) and NMDA-evoked puff fEPSPs were recorded in the *stratum radiatum* of CA1 of acute hippocampal slices at 25 °C. The recording chamber was perfused with low-magnesium ACSF containing 0.2 mM MgCl_2_, 100 µM picrotoxin and 10 µM CNQX, and gassed with 5% CO_2_/95% O_2_. Bipolar stimulation electrodes were placed among Schaffer collaterals, and glass recording electrodes (1–2 MΩ; filled with ACSF) were placed ~200 µm away in the direction of the fiber projection. A glass puff pipette (1–2 MΩ) containing NMDA (1 mM in ACSF) was positioned adjacent to the recording electrode, immediately above the surface of the slice. Responses mediated by both synaptic and extrasynaptic NMDA receptors were elicited with NMDA puffs, delivered using pressurized nitrogen (4.5 bars) gated by a Picospritzer III unit (200–500 ms pulse duration). Electrically evoked and puff fEPSPs were collected at interleaved 4-min intervals during baseline acquisition. Once a stable baseline had been recorded for 20 min, puff delivery was stopped, and MK-801 (40 µM) was infused into the bath. Stimulation frequency was subsequently increased to 0.066 Hz, and evoked responses were allowed to run down until they reached a stable nadir, at which point a final puff was delivered. Stimulation-evoked and puff fEPSPs were quantified as their peak amplitude.

### Statistical analysis

Statistical analysis was performed using the SPSS 24 software (SPSS, 1989; Apache Software Foundation, Chicago, IL, USA). The Kolmogorov–Smirnov test was used to test the normality of the sample distribution. Atypical data were detected with box-plots and eliminated when necessary. In the case of normal sample distribution, data were analyzed by a t Student test. In those cases in which normality could not be assumed, data were analyzed by the non-parametric Mann-Whitney U test. Graphs represent mean values ± SEM.

## Supplementary information

Supplementary Information

## Data Availability

The data generated during this study is included in this published article and its Supplementary File, Figs 1–5.

## References

[1] Rapoport M, Dawson HN, Binder LI, Vitek MP, Ferreira A (2002). Tau is essential to beta -amyloid-induced neurotoxicity. Proc Natl Acad Sci USA.

[2] Roberson ED (2007). Reducing endogenous tau ameliorates amyloid beta-induced deficits in an Alzheimer’s disease mouse model. Science.

[3] Shipton OA (2011). Tau protein is required for amyloid {beta}-induced impairment of hippocampal long-term potentiation. J Neurosci.

[4] Ittner LM (2010). Dendritic function of tau mediates amyloid-beta toxicity in Alzheimer’s disease mouse models. Cell.

[5] Hardingham GE, Bading H (2010). Synaptic versus extrasynaptic NMDA receptor signalling: implications for neurodegenerative disorders. Nat Rev Neurosci.

[6] Parsons MP, Raymond LA (2014). Extrasynaptic NMDA receptor involvement in central nervous system disorders. Neuron.

[7] Bi M (2017). Tau exacerbates excitotoxic brain damage in an animal model of stroke. Nat Commun.

[8] Holth JK (2013). Tau loss attenuates neuronal network hyperexcitability in mouse and Drosophila genetic models of epilepsy. J Neurosci.

[9] Gheyara AL (2014). Tau reduction prevents disease in a mouse model of Dravet syndrome. Ann Neurol.

[10] Pallas-Bazarra N (2016). Novel function of Tau in regulating the effects of external stimuli on adult hippocampal neurogenesis. EMBO J.

[11] Cheng JS (2014). Tau reduction diminishes spatial learning and memory deficits after mild repetitive traumatic brain injury in mice. PLoS One.

[12] Gladding CM, Raymond LA (2011). Mechanisms underlying NMDA receptor synaptic/extrasynaptic distribution and function. Mol Cell Neurosci.

[13] Yi Z (2007). The role of the PDZ protein GIPC in regulating NMDA receptor trafficking. J Neurosci.

[14] Goebel-Goody SM, Davies KD, Alvestad Linger RM, Freund RK, Browning MD (2009). Phospho-regulation of synaptic and extrasynaptic N-methyl-d-aspartate receptors in adult hippocampal slices. Neuroscience.

[15] Chung HJ, Huang YH, Lau LF, Huganir RL (2004). Regulation of the NMDA receptor complex and trafficking by activity-dependent phosphorylation of the NR2B subunit PDZ ligand. J Neurosci.

[16] Fulga TA (2007). Abnormal bundling and accumulation of F-actin mediates tau-induced neuronal degeneration *in vivo*. Nat Cell Biol.

[17] Regan P (2015). Tau phosphorylation at serine 396 residue is required for hippocampal LTD. J Neurosci.

[18] Whiteman IT, Minamide LS, Goh de L, Bamburg JR, Goldsbury C (2011). Rapid changes in phospho-MAP/tau epitopes during neuronal stress: cofilin-actin rods primarily recruit microtubule binding domain epitopes. PLoS One.

[19] Papouin T (2012). Synaptic and extrasynaptic NMDA receptors are gated by different endogenous coagonists. Cell.

[20] Harris AZ, Pettit DL (2007). Extrasynaptic and synaptic NMDA receptors form stable and uniform pools in rat hippocampal slices. J Physiol.

[21] Petralia RS (2010). Organization of NMDA receptors at extrasynaptic locations. Neuroscience.

[22] Roche KW (2001). Molecular determinants of NMDA receptor internalization. Nat Neurosci.

[23] Ittner A, Ittner LM (2018). Dendritic Tau in Alzheimer’s Disease. Neuron.

[24] Won S, Incontro S, Nicoll RA, Roche KW (2016). PSD-95 stabilizes NMDA receptors by inducing the degradation of STEP61. Proc Natl Acad Sci USA.

[25] Correas I, Padilla R, Avila J (1990). The tubulin-binding sequence of brain microtubule-associated proteins, tau and MAP-2, is also involved in actin binding. Biochem J.

[26] Allison DW, Gelfand VI, Spector I, Craig AM (1998). Role of actin in anchoring postsynaptic receptors in cultured hippocampal neurons: differential attachment of NMDA versus AMPA receptors. J Neurosci.

[27] Fonseca R (2012). Activity-dependent actin dynamics are required for the maintenance of long-term plasticity and for synaptic capture. Eur J Neurosci.

[28] Lisman J (2003). Actin’s actions in LTP-induced synapse growth. Neuron.

[29] Newpher TM, Ehlers MD (2008). Glutamate receptor dynamics in dendritic microdomains. Neuron.

[30] Basu S, Lamprecht R (2018). The Role of Actin Cytoskeleton in Dendritic Spines in the Maintenance of Long-Term Memory. Front Mol Neurosci.

[31] Dioli C (2017). Tau-dependent suppression of adult neurogenesis in the stressed hippocampus. Mol Psychiatry.

[32] Velazquez Ramon, Ferreira Eric, Tran An, Turner Emily C., Belfiore Ramona, Branca Caterina, Oddo Salvatore (2018). Acute tau knockdown in the hippocampus of adult mice causes learning and memory deficits. Aging Cell.

[33] Marciniak E (2017). Tau deletion promotes brain insulin resistance. J Exp Med.

[34] Tracy Tara E., Gan Li (2017). Acetylated tau in Alzheimer's disease: An instigator of synaptic dysfunction underlying memory loss. BioEssays.

[35] Babur E (2019). Depotentiation of Long-Term Potentiation Is Associated with Epitope-Specific Tau Hyper-/Hypophosphorylation in the Hippocampus of Adult Rats. J Mol Neurosci.

[36] Ahmed T (2014). Cognition and hippocampal synaptic plasticity in mice with a homozygous tau deletion. Neurobiol Aging.

[37] Didonna Alessandro, Cantó Ester, Shams Hengameh, Isobe Noriko, Zhao Chao, Caillier Stacy J., Condello Carlo, Yamate-Morgan Hana, Tiwari-Woodruff Seema K., Mofrad Mohammad R.K., Hauser Stephen L., Oksenberg Jorge R. (2019). Sex-specific Tau methylation patterns and synaptic transcriptional alterations are associated with neural vulnerability during chronic neuroinflammation. Journal of Autoimmunity.

[38] Dawson HN (2001). Inhibition of neuronal maturation in primary hippocampal neurons from tau deficient mice. J Cell Sci.

[39] Gardoni F (2009). Decreased NR2B subunit synaptic levels cause impaired long-term potentiation but not long-term depression. J Neurosci.

[40] Bai F, Witzmann FA (2007). Synaptosome proteomics. Subcell Biochem.

[41] Gylys KH, Fein JA, Yang F, Cole GM (2004). Enrichment of presynaptic and postsynaptic markers by size-based gating analysis of synaptosome preparations from rat and human cortex. Cytometry A.

[42] Llorens-Martin M (2013). GSK-3beta overexpression causes reversible alterations on postsynaptic densities and dendritic morphology of hippocampal granule neurons *in vivo*. Mol Psychiatry.

