# Supplementary material for: Tau is required for the function of extrasynaptic NMDA receptors
Source: Sci Rep. 2019 Jun 24;9:9116. doi: 10.1038/s41598-019-45547-8 (PMC6591308; doi:10.1038/s41598-019-45547-8)

Tau is required for the function of extrasynaptic NMDA receptors

Noemí Pallas-Bazarra [1,2*], Jonathan Draffin[1*], Raquel Cuadros[1,2], José Antonio Esteban[1+], Jesús Avila[1,2+]

[1] Centro de Biología Molecular Severo Ochoa (CBMSO) CSIC-UAM, Madrid, Spain.

[2] Network Center for Biomedical Research in Neurodegenerative Diseases (CIBERNED), Madrid, Spain.

* Equal Contribution

+ To whom correspondence should be addressed:

Centro de Biología Molecular Severo Ochoa, Nicolás Cabrera, 1. 28049 Madrid, Spain. Jesús Avila (e-mail: javila@cbm.csic.es Telf.:+34 91194564). José A. Esteban (e-mail: jaesteban@cbm.csic.es Telf.: +34 911964637)

E-mail address by order of all authors: n.pallas@csic.es; jdraffin@cbm.csic.es; rcuadros@cbm.csic.es; jaesteban@cbm.csic.es; javila@cbm.csic.es

SUPPLEMENTARY FIGURE

Supplementary figure 1. Absence of Tau does not affect GluN2B internalization. Western Blot (WB) analysis (left) and quantification (right) of the levels of pGluN2B(S1480) in synaptosome-enriched fractions of WT and Tau-/- mice. Graph shows mean ± SEM. N WT= 3 mice; N Tau-/- = 3 mice. The levels of pGluN2B(S1480) were calculated related to the levels of GluN2B C-ter. Actin was used as loading control. Blots were cropped to improve the clarity of the presentation. Full-length blots are presented in Supplementary figure 5.

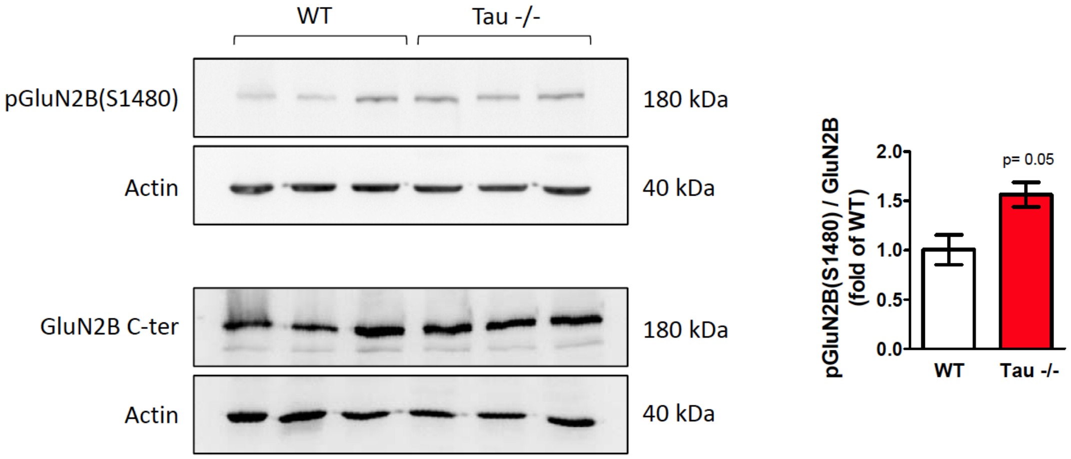

<u>Supplementary figure 2. Tau-/- mice show decreased PSD95 staining in the CA1 region of the hippocampus.</u> **(A)** Representative images of CA1 hippocampal region of WT and Tau-/- mice labeled with pGluN2B(Y1336) (green channel) and PSD95 (red channel) antibodies. **(B)** Quantification of the percentage of co-localization between pGluN2B(Y1336) and PSD95 in WT and Tau-/- mice. **(C)** Quantification of the area occupied by PSD95 in WT and Tau-/- mice. Tau-/- mice show a decrease in the area occupied by PSD95 and less co-localization between pGluN2B(Y1336) and PSD95 compared to WT ones. Given that the area occupied by pGluN2B(Y1336) does not change due to Tau absence (see figure 3), the decrease in the co-localization must be a consequence of the decrease in PSD95 area in Tau-/- mice. Graphs show mean ± SEM. N= 5 mice per genotype. Scale bar 5 µm. Brightness and contrast of representative confocal microscopy images shown in the figure were minimally adjusted in order to improve visualization.

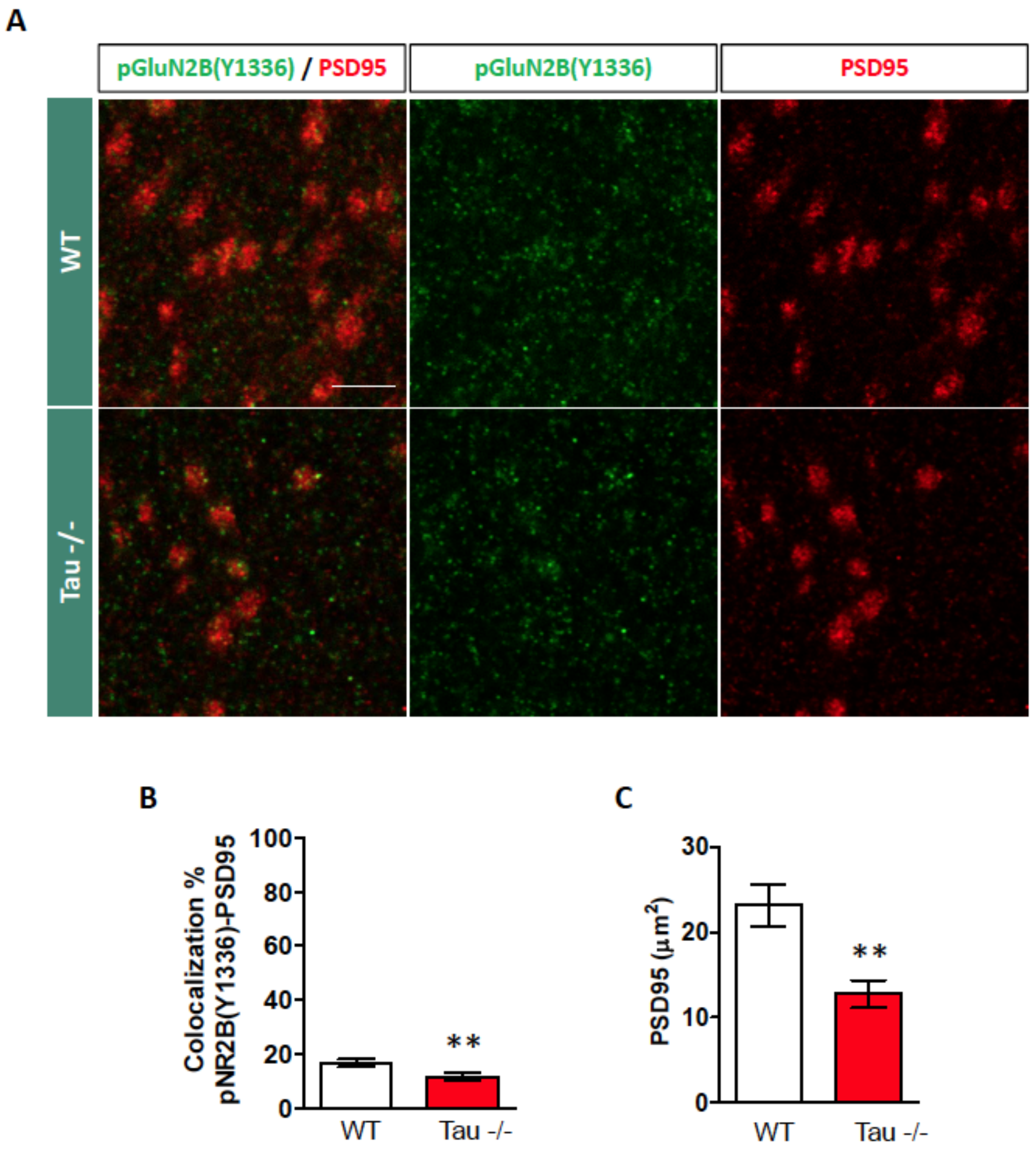

Supplementary figure 3. Tau phosphorylated at Ser 396 is present in synaptosome-enriched fractions of WT animals. Western blot (WB) analysis of pTau(S396) in hippocampal extracts and in synaptosome-enriched fractions of five WT mice. Actin was used as loading control. No reaction was found for fractions from Tau -/- mice (data not shown). Blots were cropped to improve the clarity of the presentation. Full-length blots are presented in Supplementary figure 5.

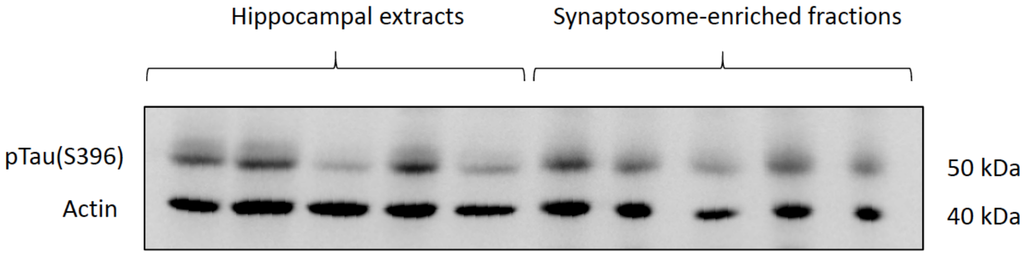

Supplementary figure 4. Enrichment in membrane-associated proteins in final synaptosome-enriched fractions obtained from subcellular fractionation of hippocampal tissue. **(A)** Western Blot (WB) analysis of the levels of PSD95 in hippocampal extracts and in final synaptosome-enriched fractions obtained from WT and Tau-/- mice. **(B)** Quantification of the rate of enrichment in PSD95 in synaptosome-enriched fractions respect to hippocampal extracts in WT and Tau-/- mice. GAPDH was used as reference protein. Graphs show mean ± SEM. Hc: hippocampal extracts. Syn: synaptosome-enriched fractions. Blots were cropped to improve the clarity of the presentation. Full-length blots are presented in Supplementary figure 5.

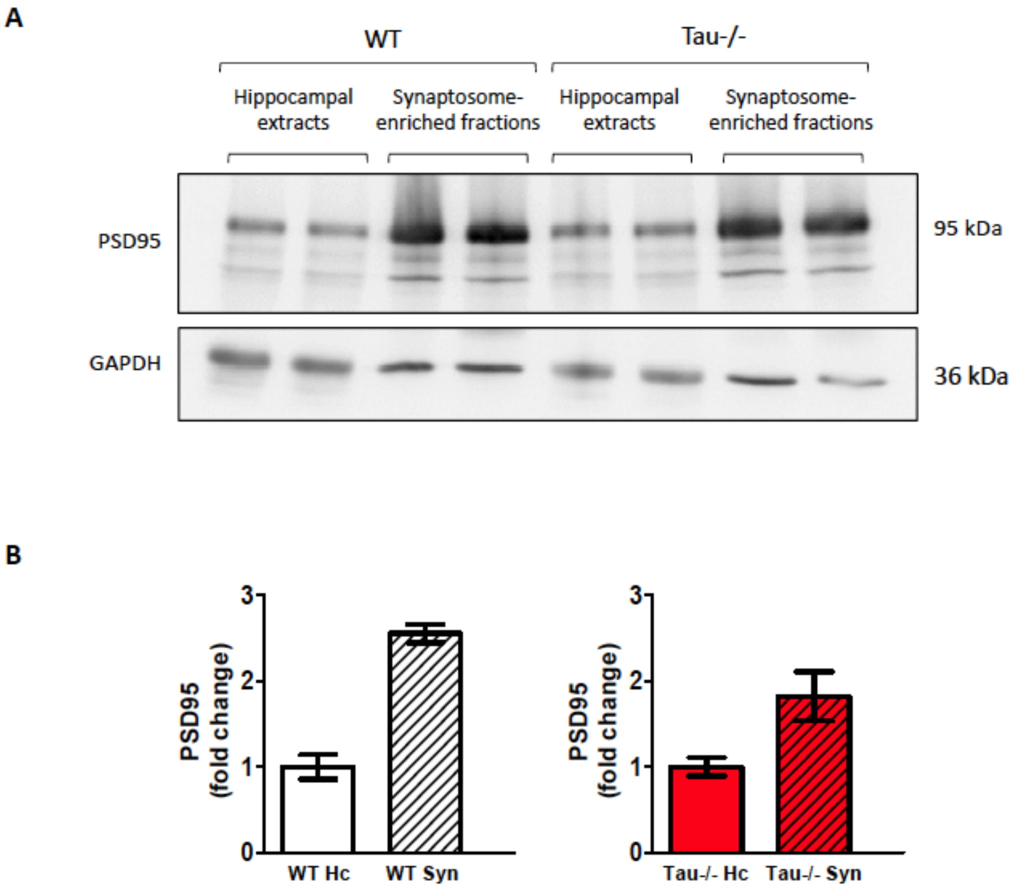

<u>Supplementary figure 5. Full-length blots.</u> Each subfigure corresponds to one blot. In Figures 1A, 1C, 2A, 2B, 2C and 2D membranes were horizontally sectioned before the incubation with the respective antibodies.

Figure 1A

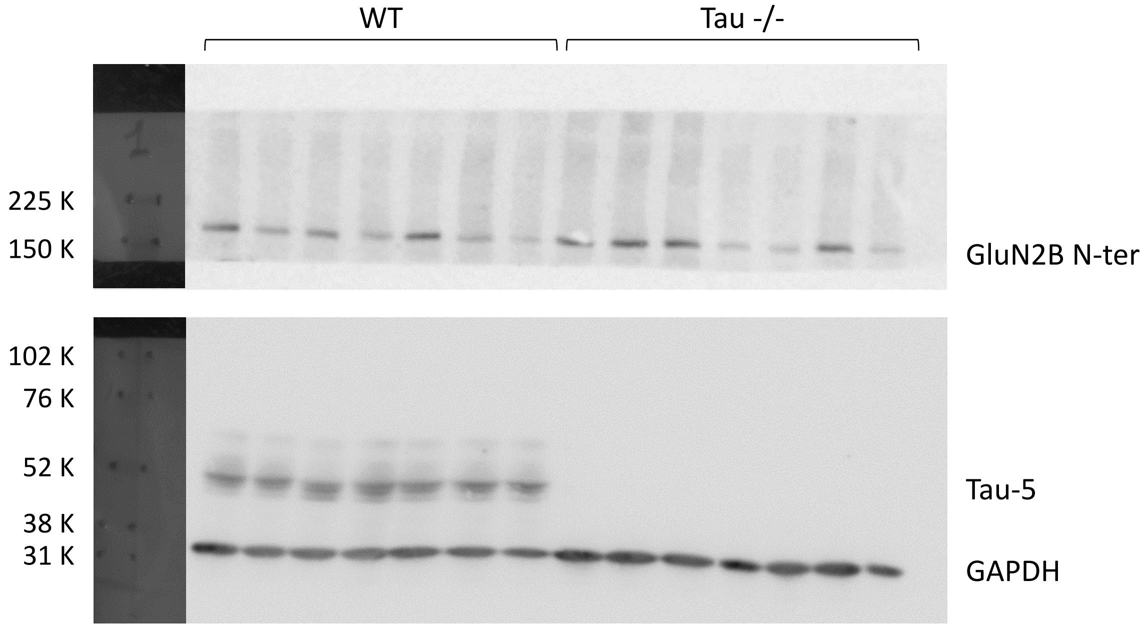

Figure 1B

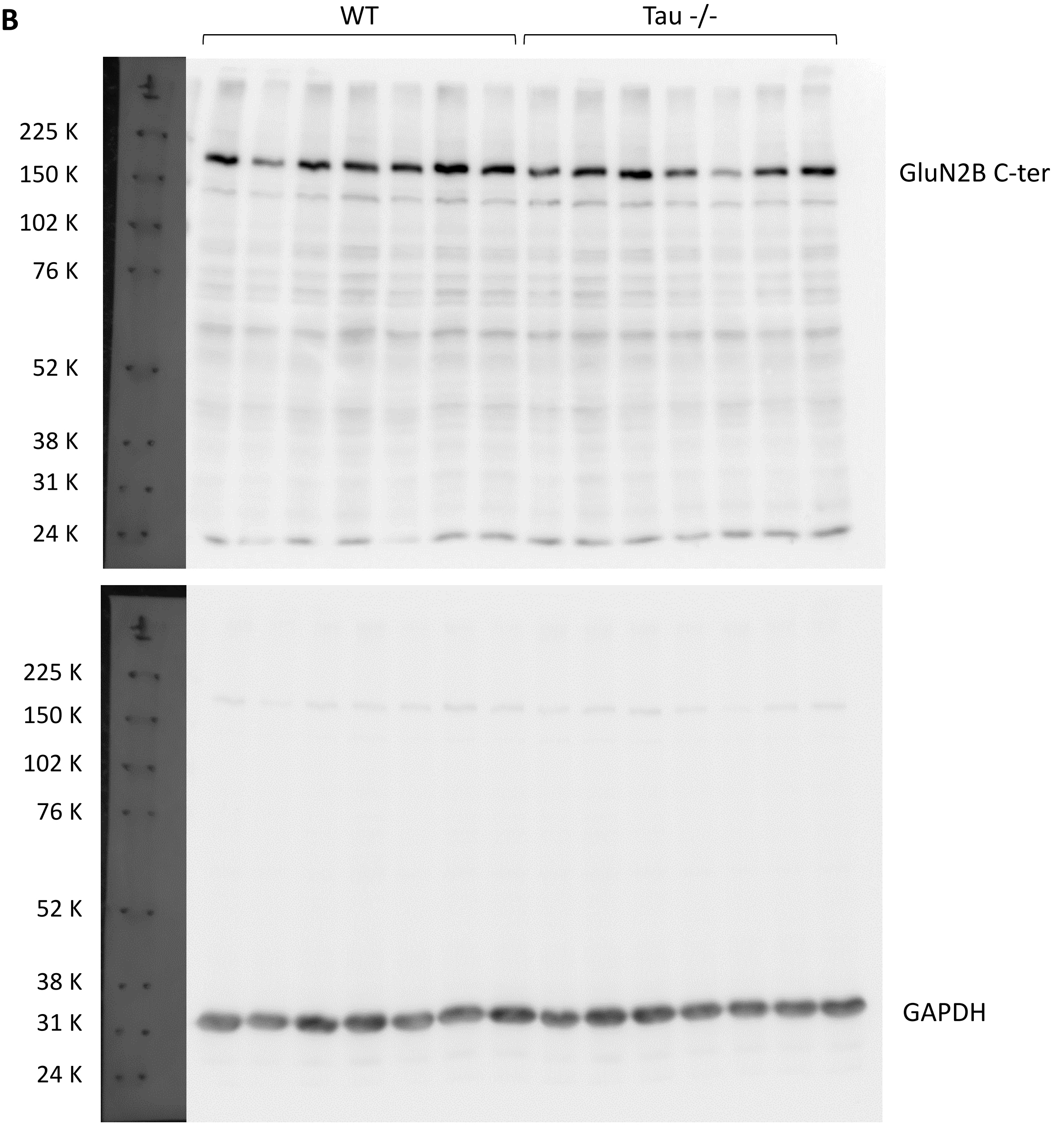

Figure 1C

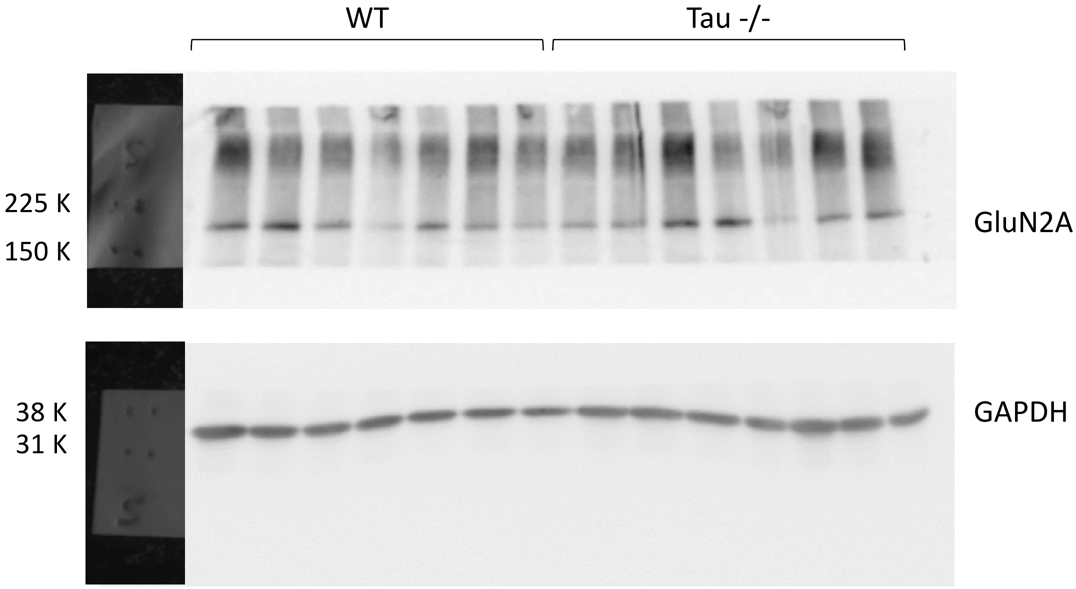

Figure 2A

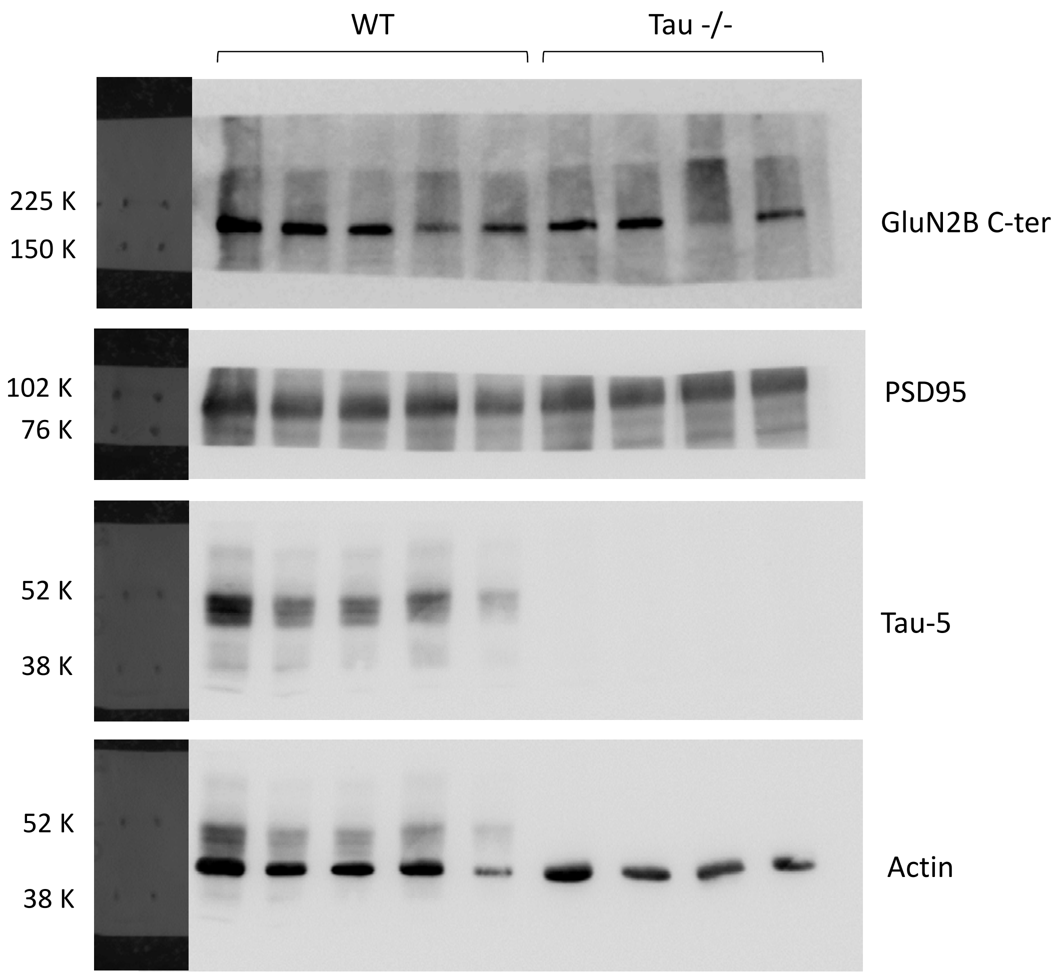

Figure 2B

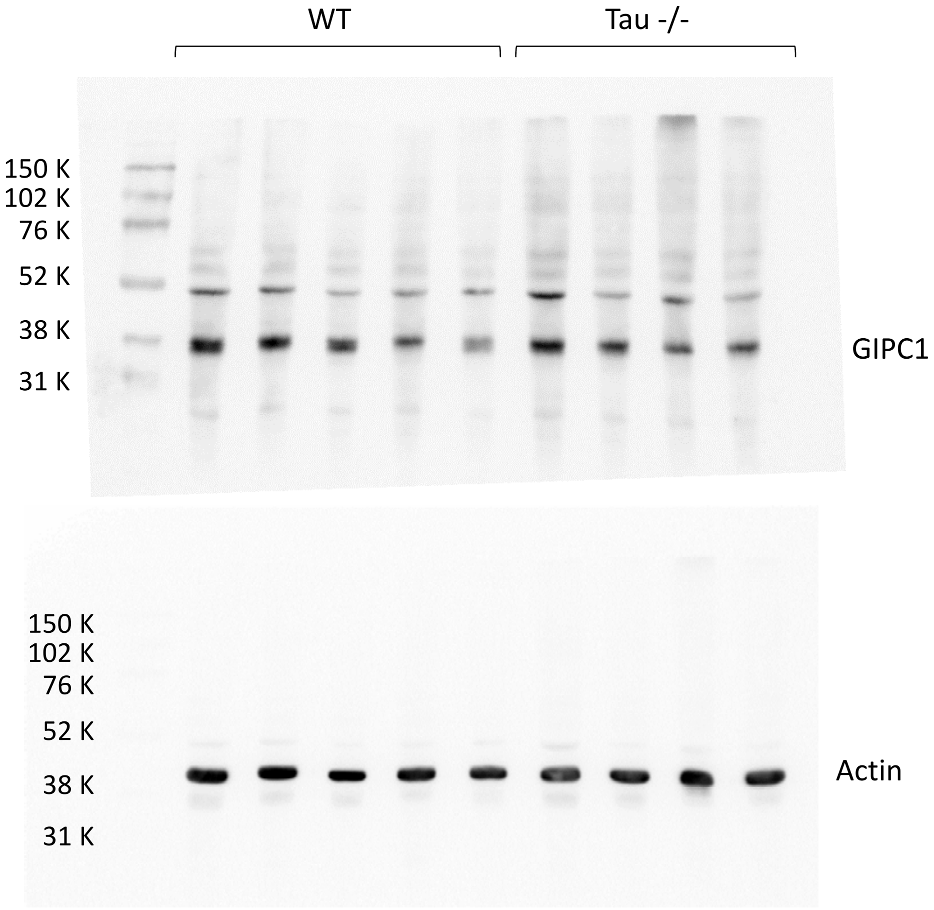

Figure 2C

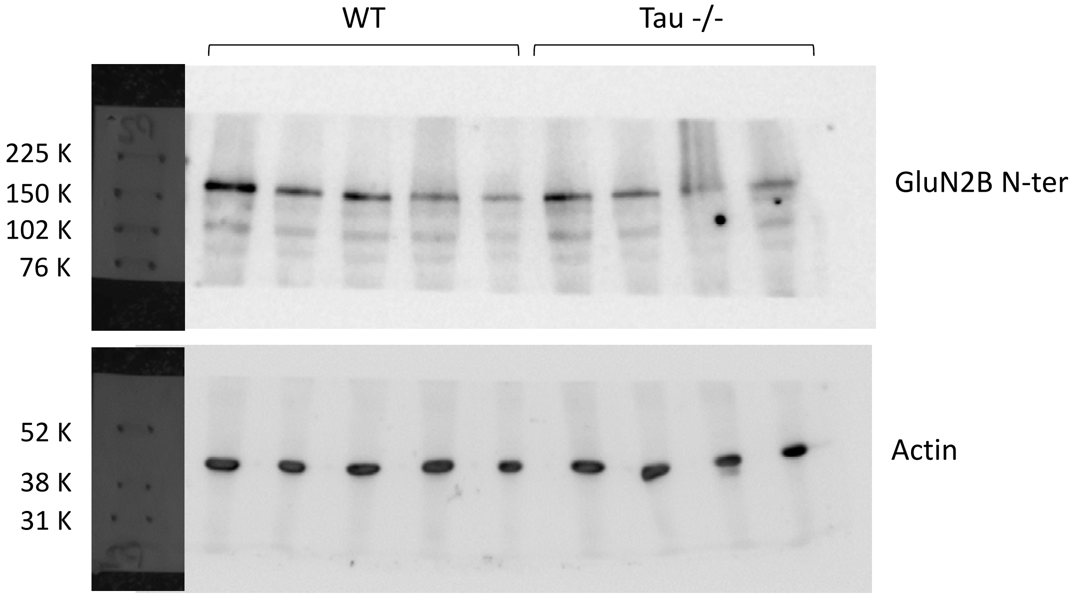

Figure 2D

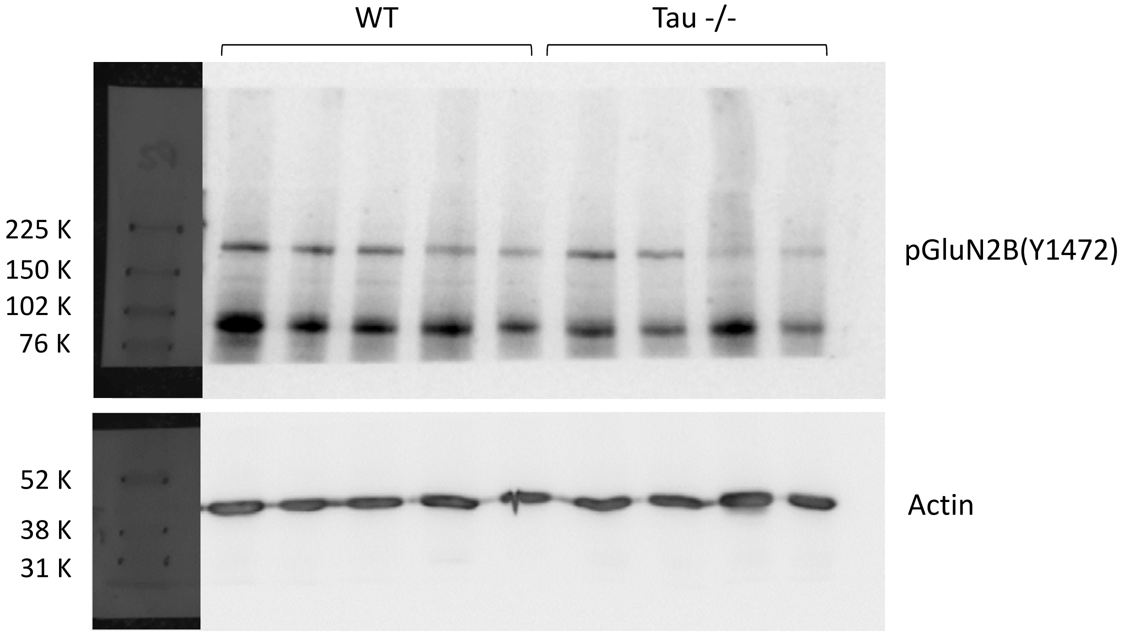

Figure 2E

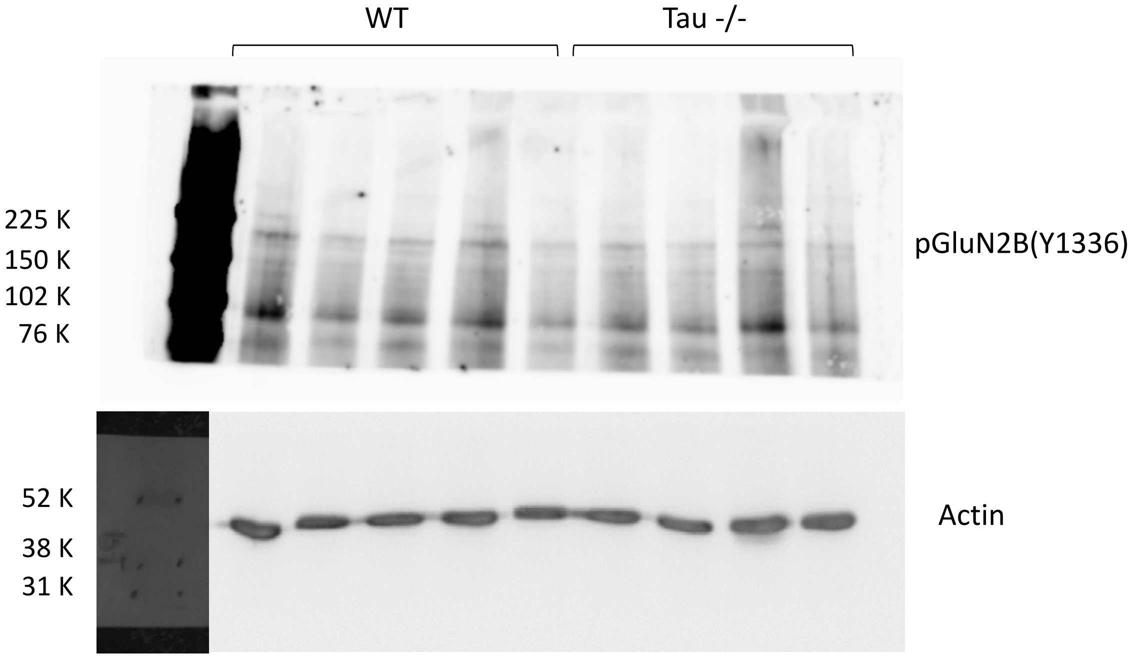

Supplementary Fig. 1

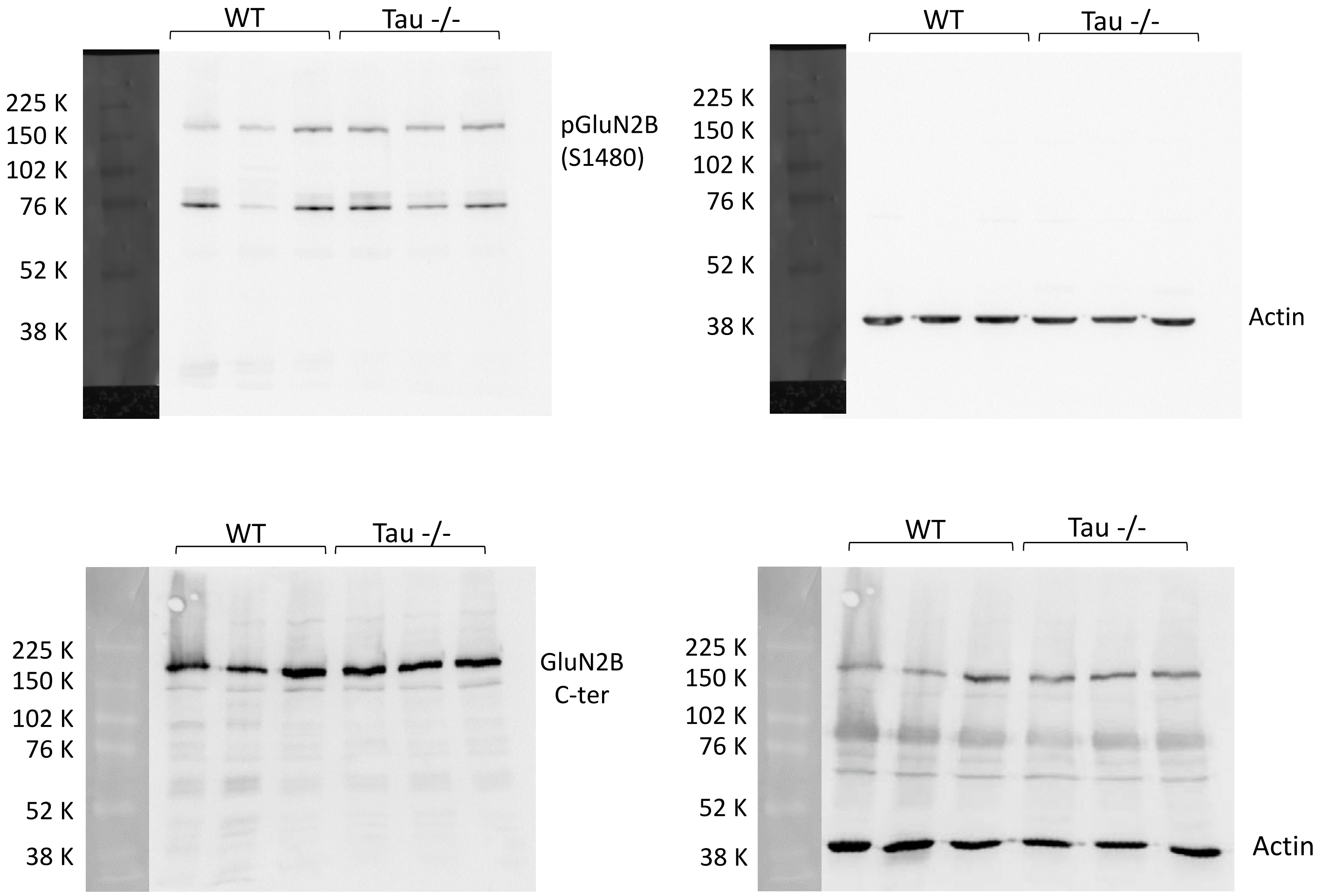

Supplementary Fig. 3

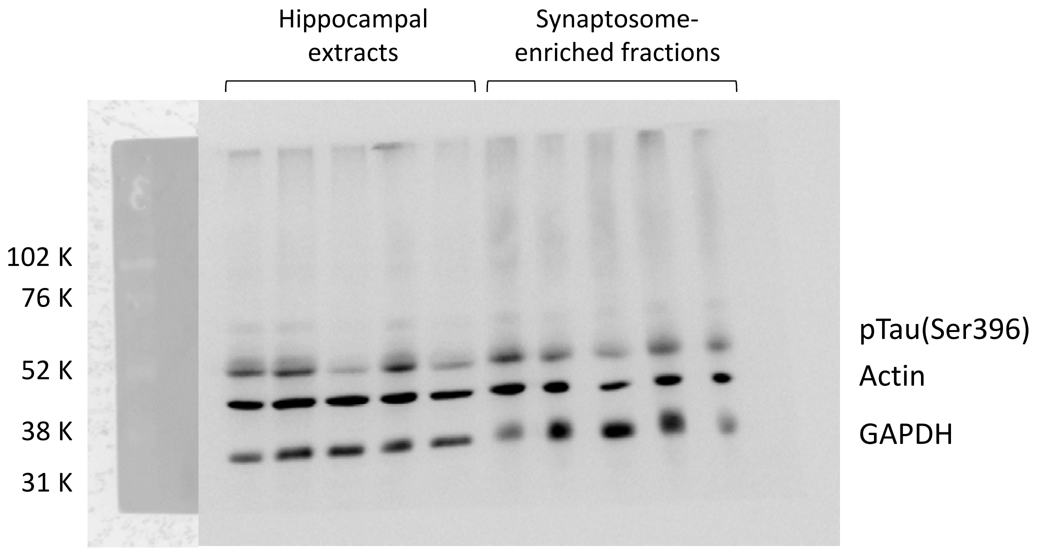

Supplementary Fig. 4

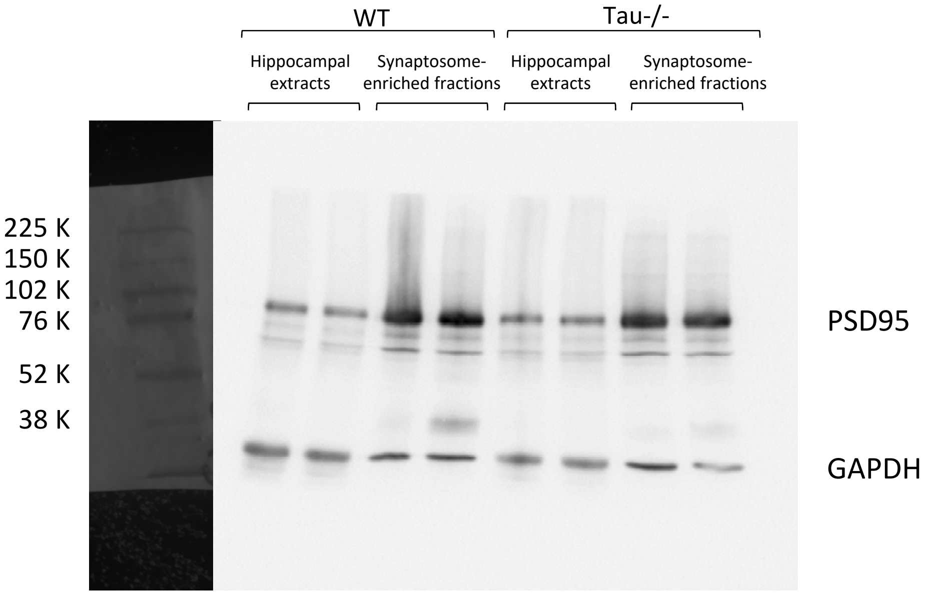

Supplement: Supplementary file 1 — Supplementary Information [file 41598_2019_45547_MOESM1_ESM.pdf]
